# Some Observations on the Local Action of Ipecacuanha

**Published:** 1874-11

**Authors:** Noel Gueneau De Mussy

**Affiliations:** Physician to the Hotel Dieu (Paris), Member of the Paris Academy of Medicine


### Some Observations on the Local Action of Ipecacuanha.

By Dr. NOEL GUENEAU DE MUSSY, Physician to the Hotel Dieu (Paris), Member of the Paris Academy of Medicine

The root of ipecacuanha has been for several centuries reputed one of the best remedies in many cases of acute dysentery, and, indeed, such faith has been put in it that the name *Radix anti-dysenterica* was one of its first appellations.

When ipecacuanha is given for dysentery, the method called Brazilian is the mode of administration which prevails generally among French physicians. They give it in small doses, boiled or infused in hot water.

In many cases, and more especially when dysentery is complicated with gastric symptoms, which is very commonly to be observed, I have found it useful to begin with liberal doses, so as to obtain the effect of an emetic, and it is not till later that I give it according to the Brazilian method, in the form of a decoction or of ipecac. syrup: one teaspoonful of the latter every two or three hours.

Lately I had under my care an American gentlemen who, in a severe attack of dysentery, was not relieved after six or seven days' use of decoction of the root mixed with opium, according to the prescription of a naval surgeon. Other kinds of treatment had also proved ineffectual; and though I found him very weak and exhausted by protracted dysentery, as he complained of nausea, and as his tongue was thickly furred, I prescribed ipecacuanha as an emetic, to be followed, after vomiting, by small doses of the syrup. This was attended by immediate relief, and the patient's health was soon restored.

In chronic dysentery, and even in common chronic diarrhœa, injections of decoction of ipecacuanha into the intestines are a common practice in Peru and in some other countries of South America.

I have used this remedy with success in some cases of diarrhœa unchecked by other means. My formula is thus: Ipecac. root, ʒi.; boil for ten minutes in water, ℥v. Let it infuse for one or two hours, strain off, and make use of the decoction as an enema.

Habitually this enema is wonderfully well tolerated. No painful sensation, no irritation of the bowel, attends these injections in the greater number of cases. They can be retained for several hours without any difficulty, and even, occasionally, with a feeling of comfort and relief.

This successful result of the local application of the decoction in enteritis, induced me to try it in some other inflammatory affections of mucous membranes.

In the beginning of the year 1872, I received into my wards a new-born female child, about eighteen days old. She looked very poorly fed, was thin and wan, and her limbs were cold and blue, though no anomaly could be detected in the central circulation. From the red closed and swollen eyelids oozed a muco-purulent matter, which, flowing on the cheek, irritated by its contact the skin around the eyes and the naso-labial grooves.

The eye-lids could only be raised with great difficulty, and, on doing so, the mucous lining would protrude outwards—scarlet-colored, swollen, velvet-like—between the streams of purulent matter which escaped from the surface of the eyeball.

The left cornea was dull, rough, deprived of its brightness and transparency. A small ulceration, of the size of a millet seed, occupied the central part of it. A light whitish cloud darkened all the surface of tha right cornea.

The child's mother was weak, anæmic, but free from any venereal contamination.

I prescribed the treatment which, for more than thirty years, I have scarcely ever found to fail in purulent ophthalmia of new-born children. An injection was ordered to be made every hour with a solution of two grains of nitrate of silver in three and a half ounces of distilled water. Four times a day, a stronger solution, containing the same quantity of the nitrate to one ounce only of water, was to be instilled.

The state of the eyes greatly improved, and the acute symptoms subsided. The purulent secretion was almost entirely dried up: but the inflammatory process was not quite extinguished. The conjunctiva remained swollen, red and slightly granulated. The cornea presented the same appearance. I touched it with a crayon composed of equal proportions of nitrate of potash and nitrate of silver, but no change took place in the condition of the affected parts. The ulceration and the opacity of both corneæ remained unmodified.

After four days of useless application of this remedy, it occurred to my mind that decoction of ipecacuanha, which had proved so useful in sub-acute inflammation of the bowels, might be successful in this case.

So I prescribed, four times daily, an instillation to be made into both eyes with the following decoction:—Ipecac. root, ʒss.; water, ℥v. Boil for ten minutes, and, when cool, strain off.

The application of this topic seemed at first rather painful; the child winked, frowned and cried after each instillation. But it soon got accustomed to them, and the affected parts were speedily modified. After twelve days the granular appearance had disappeared; the conjunctiva recovered its natural color; the right cornea was quite healthy; only slight opacity was to be observed in the left; and, after some days, the baby left the Hôtel-Dieu entirely cured.

I related this observation to my learned friend, Dr. Galezowski, who tried the remedy in the same conditions of *sub-acute* inflammation, and in several cases with success.–*The London Practitioner.*
—*Boston Med. and Surg. Jonrnal.*

